# Data-driven sequence learning or search: What are the prerequisites for the generation of explicit sequence knowledge?

**DOI:** 10.2478/v10053-008-0110-4

**Published:** 2012-05-21

**Authors:** Sabine Schwager, Dennis Rünger, Robert Gaschler, Peter A. Frensch

**Affiliations:** 1 Department of Psychology, Humboldt University of Berlin, Germany; 2 Department of Psychology, University of California, Santa Barbara, USA

**Keywords:** sequence learning, explicit sequence knowledge, sequence detection, serial reaction time task, reportable knowledge, unexpected events

## Abstract

In incidental sequence learning situations, there is often a number of participants who can report the task-inherent sequential regularity after training. Two kinds of mechanisms for the generation of this explicit knowledge have been proposed in the literature. First, a sequence representation may become explicit when its strength reaches a certain level (Cleeremans, 2006), and secondly, explicit knowledge may emerge as the result of a search process that is triggered by unexpected events that occur during task processing and require an explanation (the unexpected-event hypothesis; Haider & Frensch, 2009). Our study aimed at systematically exploring the contribution of both mechanisms to the generation of explicit sequence knowledge in an incidental learning situation. We varied the amount of specific sequence training and inserted unexpected events into a 6-choice serial reaction time task. Results support the unexpected-event view, as the generation of explicit sequence knowledge could not be predicted by the representation strength acquired through implicit sequence learning. Rather sequence detection turned out to be more likely when participants were shifted to the fixed repeating sequence after training than when practicing one and the same fixed sequence without interruption. The behavioral effects of representation strength appear to be related to the effectiveness of unexpected changes in performance as triggers of a controlled search.

## Introduction

Everyday life offers many opportunities to learn about environmental regularities. It is likely that a large part of this learning is not driven by an explicit intention to learn. A strong example of the latter possibility is the acquisition of one’s native language, which occurs at an age when explicit learning strategies are not yet available and grammatical rules cannot be reported. Therefore, it may be argued that in many cases learning takes place implicitly. People neither have an intention to learn, nor do they necessarily become aware of the regularities they have acquired (cf. Frensch, 1998). Most action sequences (motor as well as cognitive) are probably learned this way: “by doing” and without top-down control through a declarative representation of the regularity underlying the composition of the task material.

A widely used experimental paradigm to investigate incidental learning is the serial reaction time (SRT) task, first introduced by Nissen and Bullemer (1987). In this task participants have to respond to the location of an asterisk on the computer screen by pressing one of four response keys. The key feature of the task is that target locations on consecutive trials are predetermined and follow a repeating pattern. There is ample evidence that such spatio-temporal relations between successive events can be learned and that they can influence task performance, even when participants find it difficult or impossible to describe the regularity verbally (for overviews, see e.g., Frensch & Rünger, 2003; Shanks, 2005; Stadler & Frensch, 1998). Therefore, when learning happens without the explicit intention to learn (cf. e.g., the definition of *incidental learning* in Frensch, 1998) the acquired know-ledge is often implicit. On the other hand, many studies in the implicit learning literature report that at least some participants acquire explicit knowledge about the hidden regularity in a sequence learning task (e.g., Buchner, Steffens, Erdfelder, & Rothkegel, 1997; Zirngibl & Koch, 2002). Moreover, profound performance gains have been linked to awareness of task regularities (e.g., Haider, Frensch, & Joram, 2005; Rünger & Frensch, 2008; Tubau, Hommel, & López-Moliner, 2007), and there is evidence that people (e.g., Gaissmaier & Schooler, 2008) and even rats (e.g., Harlow, 1949; Tolman, 1948), under some circumstances, spontaneously engage in an active search for environmental regularities. This raises the question of how and when people become aware of these regularities. Two theoretical accounts can be distinguished that either emphasize the role of a continuous strengthening of memory representations or propose the idea of explicit hypotheses testing in the generation of explicit knowledge.

According to the first theoretical account (Cleeremans, 2006; Cleeremans & Jiménez, 2002), learning is a mandatory consequence of task processing. The quality of a memory representation - its stability, strength, and distinctiveness - increases gradually over the course of learning. Quality, in turn, determines the influence of a representation on behavior as well as its availability to consciousness and to intentional control. Once a representation enters awareness by dint of its high quality, other controlled operations (such as recoding into linguistic propositions and the generation of metaknowledge) become possible. Importantly, Cleeremans and Jiménez (2002) posit a direct relation between the gradually increasing strength of the memory representation and the emergence of explicit knowledge. In an incidental learning situation, repeated exposure to an environmental regularity gradually strengthens representations that support behavioral adaptation to the regularity. A sufficiently strong representation of this regularity enables the individual to verbally report the regularity and to use this know-ledge to perform the task at hand more efficiently.

According to the second theoretical approach (see Frensch et al., 2003; Haider & Frensch, 2005, 2009; cf. also Clapper & Bower, 2002; Sun, Merrill, & Peterson, 2001), there is no such direct relation between the quality or strength of memory representations acquired through incidental learning and conscious awareness of the regularity. According to the so-called unexpected-event hypothesis (see Frensch et al., 2003; Haider & Frensch, 2005, 2009), explicit knowledge about an incidentally experienced regularity is generated by a controlled search in addition to regular task processing. This search is triggered by unexpected events that occur during task processing and call for an explanation. While performing an incidental sequence learning task, for instance, subjects may experience an unexpected feeling of fluency that does not correspond to the perceived task difficulty, and while searching for the origin of the unexpected fluency they find the regular pattern built into the task.

Support for the unexpected-event hypothesis comes from a study by Rünger and Frensch (2008). They conducted a series of experiments with the SRT task in which they tested the impact of changes in the sequential structure on the likelihood to develop verbalizable sequence knowledge. Compared to a condition with no change in sequence structure, they found that more participants acquired explicit sequence knowledge when they repeatedly transitioned back and forth between two different systematic sequences. The authors assumed that the shifts functioned as unexpected events. Presumably, participants had adapted to the SRT task by implicitly learning the systematic patterns. Therefore, shifts from one fixed sequence to the other should have disrupted participants’ performance. In search for the causes of these unexpected changes in their behavior, participants were then likely to discover the repeating sequence structure(s). Haider and Frensch (2009) manipulated the occurrence of unexpected events more directly and demonstrated that artificially induced (computer generated) premature responses can increase the availability of reportable knowledge about a task regularity.

Assuming explicit knowledge to be the result of a controlled search implies that (a) unexpected events (and the subsequent search process) do not have to be a direct consequence of implicit learning, and (b) likewise, that the result of the search needs not be related to the specific implicit representation and its strength. Thus, the unexpected-event hypothesis and Cleereman’s memory-strength account differ in how the link between implicit learning and the generation of explicit knowledge is conceptualized. According to Cleeremans (2006), the distinction between implicit and explicit knowledge is a matter of representation strength, while Frensch and collaborators (2003) posit dedicated memory systems for implicit and explicit learning.

The aim of the present study was to examine the role of an incidentally acquired sequence representation and of the occurrence of unexpected events in the generation of explicit, verbalizable sequence knowledge. Specifically, we wanted to know if the occurrence of explicit sequence knowledge is determined by the increasing strength of an implicit sequence representation, or if it is, at least to some extent, independent of representation strength.

### Experimental approach

In our experiment, we scrutinized two ideas: (a) the assumption that increasing the strength of a sequence representation increases the probability of generating verbalizable sequence knowledge, and (b) the possibility that unexpected changes in one’s performance trigger a controlled search for the cause of these changes that may lead to explicit sequence knowledge. With regard to the first issue, we experimentally manipulated the amount of practice with a repeating sequence. To investigate the second issue, we focused on participants’ expectations about the timing of events. Rünger and Frensch (2008) sought to induce unexpected changes in task performance by shifting participants repeatedly between two different sequential regularities. In the present study, we took a more direct approach and induced deviations from the expected timing of events by manipulating the response-stimulus interval (RSI).

Participants performed a modified version of the SRT task with a repeating six-element first order conditional (FOC) sequence (cf. Reed & Johnson, 1994). In all experimental conditions, 300 training trials were followed by a manipulation phase that consisted of 180 trials (see Table 1.).

**Table 1.** Overview of the Five Experimental Conditions.

Experimental group	Training phase (300 trials)	Manipulation phase (180 trials)
Random_C_	Random sequence	Regular sequence
Sequence_C_	Regular sequence	Regular sequence
Random_RSI_	Random sequence	Regular sequence^a^
Sequence_RSI_	Regular sequence	Regular sequence^a^
Sequence_T_	Regular sequence	New regular sequence

RSI = response-stimulus interval. C = control. T = transfer. ^a^ RSI was shorted in 18 trial triplets.

Two groups practiced the task with random material before being exposed to the systematic sequence. For three groups, the task started with a repeating sequence. While this sequence continued throughout the whole experiment for two of these groups, the third group was transferred to a different repeating sequence during the final 180 trials. If the strength of the sequence representation plays a pivotal role in the generation of explicit sequence knowledge, more participants should acquire explicit knowledge in the groups with sequence training than in the groups with random training. Crucially, according to the account of Cleeremans and collaborators (Cleeremans, 2006; Cleeremans & Jiménez, 2002), this should hold for the two groups that were exposed to just one regular sequence throughout the experiment, but not for participants that were shifted to a different fixed sequence in the manipulation phase. In their view, the representation of a specific sequence becomes available to consciousness due to continuously operating learning mechanisms, it reaches a sufficient level of quality or strength. Prior strengthening of a different sequence representation should hinder rather than help the generation of awareness of the systematic sequence introduced in the final phase of the experiment. According to the *unexpected-event hypothesis*, however, a shift from one systematic pattern to a different fixed sequence can increase the chance that verbalizable knowledge is generated.

In order to investigate the effects of unexpected events with regard to timing, we introduced deviations from the standard RSI in two experimental groups. For one of the groups with random training and one of the groups exposed to a single repeating sequence, the timing manipulation was introduced in the last 180 trials. We reasoned that if unexpected changes in the perceived timing of task performance can trigger the generation of explicit sequence knowledge, more participants should be able to verbalize the sequence in the groups with the timing manipulation than in the respective control groups without a timing manipulation.

We conducted a pilot experiment to determine the magnitude of our RSI manipulation. Sixteen participants performed 15 blocks of the same six-choice SRT task that was used in the current experiment. Each block contained four different RSI manipulations (each once) at randomly selected positions within the block: RSI was shortened on one trial by 100 ms relative to the standard RSI of 400 ms, and on one trial by 200 ms. One triplet of consecutive trials was presented with an RSI shortened by 100 ms, and one triplet of trials with an RSI shortened by 200 ms. The question “Was the last trial faster than usual?” (German: “War das Tempo zuletzt schneller?”) was displayed. We found that participants were most likely to experience a relative increase in speed when the RSI was shortened by 200 ms on three consecutive trials. The mean probability of indicating an “increased tempo” was 51% after trial triplets deviating by 200 ms (32% after comparable trials with standard RSI, 35% after triplets deviating by 100 ms, 38% after single 200 ms-deviants, and 37% after single 100 ms-deviants).

Deviations in RSI are not the only potential source of unexpected events. In line with previous findings by Rünger and Frensch (2008), we assumed that the transition from one repeating sequence to another provides a different means of inducing unexpected events. Responding to targets that follow a sequence different to the one that was learned implicitly should lead to an increase in reaction time (RT). In contrast to the unexpected speed-up induced by the RSI manipulation, participants should experience an unexpected slowing of their responses. An increased number of participants who acquired explicit sequence knowledge after being transferred to a novel sequence would support the notion that the effectiveness of unexpected events related to one’s own motor performance is not restricted to the specific sequence re-presentation acquired during training. Since the slowing of responses after a pattern shift should occur continuously over several trials, this manipulation might be even more effective than artificially inducing an unexpected speed-up in a limited number of trials.

## Method

### Participants

We recruited 284 participants (*M*_age_= 24.9, **SD** = 4.22), predominantly students at Berlin universities, to take part in the experiment. They were paid 4 € for participation. Thirty-two participants had to be excluded from the main analyses because they either reported that they had participated in a similar (incidental learning) experiment before or already expected to encounter some form of hidden regularity before they even started to perform the SRT task. The remaining participants, 152 women and 100 men, were assigned to the five experimental conditions. Thirty-four women and 25 men made up the control group with random training (Random_C_), 21 women and 16 men made up the group with random training and a timing manipulation during the last 180 trials (Random_RSI_), 41 women and 15 men comprised the control group with a repeating sequence from the beginning of training (Sequence_C_), 31 women and 25 men comprised the group with sequence training and timing manipulation (Sequence_RSI_), and 25 women and 19 menmade up the group with sequence training and an alternate sequence in the final 180 trials (Sequence_T_).

### Apparatus

Stimulus presentation, RT measurement, and response recording were implemented on IBM compatible PCs with 33 cm color monitors and standard German QWERTZ keyboards. The viewing distance was approximately 60 cm. A large colored rectangle (8 cm wide and 6 cm high) and six small colored squares (side length = 2.5 cm) were displayed simultaneously on a light gray background. The large rectangle was centered in the top half of the display, 3 cm below the top of the monitor. The six small squares, subsequently referred to as Target Squares 1 to 6, appeared 3.5 cm from the bottom of the monitor and 9 cm below the top rectangle. They were separated horizontally by 2 cm, except for the third and fourth squares, which were spaced 3 cm apart. Each target square was mapped to a spatially compatible response key on the computer keyboard: [*X*],[*C*], [*V*], [*B*], [*N*], and [*M*]. The response keys were labeled *1* to *6* from left to right. The same six colors (green, red, cyan, dark gray, magenta, and blue) were used on every trial, but each square changed its color pseudorandomly from one trial to the next.

### Materials

In all conditions (sequence and random training conditions) response locations were governed by a repeating six-element FOC sequence during the last 180 trials with the SRT task. Each of the six possible response locations occurred once in the sequence (e.g., “152643”). Consequently, the response location on any given trial was predictive of the response location on the next trial. Our SRT task contained no further sequential regularities other than the repeating sequence of response locations. Each participant was randomly assigned to a six-element sequence that was drawn from a pool of 70 sequences. The sequences were permutations of the six response locations that satisfied the following conditions: First, “runs” of three or more adjacent response locations (e.g., “123,” “2345,” “654”) were not permitted. Second, adjacent response locations (e.g., “12,” “34,” “65”) could not occur more than twice within a sequence. We employed a six-element first order conditional sequence because prior works (e.g., Rünger & Frensch, 2008) indicated that such a sequence can be discovered relatively easily, if one searches for a regularity. A longer sequence or a sequence with fewer response alternatives that includes second order transitions would likely lead to the development of partial explicit knowledge in many participants. In contrast, previous studies in our lab showed that fixed sequences of six responses produce bimodal distributions. After the training phase, the majority of participants were able to verbalize either the whole sequence or nothing at all.

Participants in the conditions with sequence training received the repeating sequence of response locations from the beginning. In the Sequence_T_ group, response locations in the final 180 trials followed a different repeating sequence that was selected pseudorandomly from the pool of 70 sequences with the constraint that the second sequence could not share any transitions between adjacent sequence elements with the training sequence. For example, if response location “2” preceded response location “1” in the training sequence, then response location “2” had to be followed by a location other than “1” in the transfer sequence. In the training phase of the Random groups, response locations occurred randomly with the constraint that repetitions were not allowed.

### Procedure

Participants were told that they were taking part in a simple choice RT experiment designed to see how practice affects the ability to discriminate colors. They were not informed of the fact that correct response locations could follow a repeating pattern. Learning of the sequential regularity was thus incidental. Instructions for the SRT task were presented onscreen in the presence of the experimenter and followed by 40 warm-up trials during which response locations were determined randomly with the constraint that a response location could not be used on consecutive trials. The warm-up trials were repeated if a participant made mistakes on more than 20% of the trials. Response locations during the training phase (first 300 of 480 trials) in the Random groups were determined in the same manner as the warm-up trials.

The experiment comprised four blocks, during which participants performed the six-choice color matching version of the SRT task. Each block consisted of 120 trials, for a total of 480 trials. The Random_C_ and Random_RSI_ groups performed 30 repetitions of the six-element FOC sequence in their last one and a half experimental blocks, and 300 randomly sequenced trials in their first two and a half blocks. The Sequence_C_ and Sequence_RSI_ groups performed a total of 80 sequence repetitions throughout Blocks 1 to 4. On each trial, participants had to determine which of the six target squares at the bottom of the screen matched the color of the large rectangle on top and to press the response key that was assigned to that target square. They responded to Target Squares 1, 2, and 3 with the ring, middle, and index fingers of their left hands, and to Target Squares 4, 5, and 6 with the index, middle, and ring fingers of their right hands, respectively.

The first target location in each trial block was determined randomly with the constraint that the response location had to differ from the response location on the final trial of the previous block. Thereafter, response locations were chosen according to sequential regularity or randomly in the first two and a half blocks of groups with random training. A trial ended when a participant pressed one of the six response keys. In the case of an erroneous response, participants heard a beep for a duration of 100 ms. When the response key was released, the screen blanked after 200 ms, and the next trial began 200 ms later. The total RSI was therefore 400 ms. Response latencies were measured from the onset of a trial to the depression of the response key. Participants received feedback about their mean RTs and error rates after each block of 120 trials. If the error rate exceeded 10%, participants were prompted to make fewer mistakes.

During the last one and a half experimental blocks in the Random_RSI_ and Sequence_RSI_ groups, the RSI was shortened by 200 ms in 30% of the trials in the following way. The screen blanked immediately after the response, and the next stimulus occurred 200 ms later (resulting in an RSI of 200 ms in contrast to the standard RSI of 400 ms) on three consecutive trials. These 18 triplets of trials were placed quasi randomly within the sequence of trials with two constraints: Between two trial triplets with shortened RSI, at least three trials with standard RSI had to be presented, and the starting trials of the triplets had to meet each position within the repeating response sequence equally often.

Upon completion of the final block of trials, the experimenter returned to the testing cubicle and assessed participants’ reportable knowledge about the sequence presented in the manipulation phase in a semi-structured interview. The experimenter presented a cue card with six boxes labeled 1 to 6 and told the participant that the boxes represented the six response keys that corresponded to the six target squares. The experimenter then declared that responses in the final one and a half experimental blocks followed a regular pattern and asked the participant to verbally describe the serial order of response locations by referring to the labels on the cue card. In order to prevent any spontaneous typing activity, we asked participants to cross their arms in front of their upper body and hold a pencil in each hand while they attempted to report the sequence of response locations. Note that we deliberately deviated from the common strategy of opening the assessment of verbalizable sequence knowledge with general questions about the task as, in our view, a clear focus on the relevant serial-order information ensures maximum sensitivity of the verbal report measure (Rünger & Frensch, 2010).

In line with the theoretical focus on the generation of verbalizable knowledge in an incidental learning task (cf. Rünger & Frensch, 2010), our dependent measure was the free verbal report described above. In order to gain exploratory evidence on the relative sensitivity of cued verbal report, we additionally assessed it as a second measure. As this was done after the free verbal report, reactive effects of the first test of explicit sequence knowledge are possible. However, as subsequent administration of both tests seems to be the only way to gain any information on the correlation of the respective measures, we decided to include the cued test nevertheless. For the cued recall test, the experimenter named the six response positions in random order and participants were asked to indicate the two following response positions in each case. After each answer, they provided a confidence judgment. After this cued recall test, participants who verbalized a sequence at the beginning of the interview were asked in which block of the experiment they detected it. A final question prior to debriefing concerned any preexisting notions regarding the purpose of the experiment and, in particular, regarding hidden regularities. If a participant indicated a priori expectations about a hidden regularity, he or she was excluded from further analyses.

## Results

### Evaluation of explicit sequence knowledge

We focused on free verbal report (as described above) as assessment of explicit sequence knowledge and will report on cued verbal report at the end of the Results section. Participants were categorized as “verbalizers” if they correctly reported at least four consecutive elements of the sequence that was presented in the final 180 trials of the experiment. In a previous work, Rünger and Frensch (2008) estimated the probabilities of reporting the entire sequence or parts of the sequence by mere guessing. The probability of producing a correct quadruple by guessing was determined to be less than 3% (see Rünger & Frensch, 2008, p. 1016). This corresponds to our observation that participants who verbalized at least one correct quadruple also reported that they became aware of the response sequence during the experiment and reproduced it in a fluent manner, typically swapping two adjacent elements in the case of an incorrect report. Therefore, we decided to use this dichotomous measure (proportion of verbalizers as an estimate for the probability to generate explicit sequence knowledge) for comparisons between experimental conditions rather than calculating a group average of the raw verbal report data.

### Overall proportion of verbalizers

**Figure 1.**
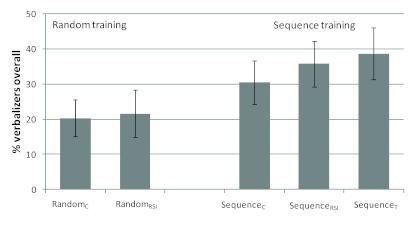
Percentage of participants categorized as “verbalizers” in the five experimental groups with randomized and systematic training. In the manipulation phase, all groups received a systematic sequence which was the same as before in the Sequence_C_ and Sequence_RSI_ groups and a new one in the Sequence_T_ group. The manipulation phase of the RSI groups additionally contained shortened RSI triplets. Error bars represent estimated standard errors for percent values. RSI = response-stimulus interval. C = control. T = transfer.

The proportion of verbalizers was 20.3% in the Random_C_ condition, 21.6% in the Random_RSI_ condition, 30.4% in the Sequence_C_ condition, 35.7% in the Sequence_RSI_ condition, and 38.6% in the Sequence_T_ condition (see Figure 1). Before turning to the four conditions that crossed the factors RSI and sequence training, we evaluated the Sequence_T_ condition. Participants in this condition outperformed participants in the other conditions numerically. First, from the standpoint that representation strength accumulates for a specific systematic sequence until it becomes verbalizable (Cleeremans & Jiménez, 2002), the Random_C_ condition can serve as a baseline. Participants in the Sequence_T_ and the Random_C_ condition received the same amount of training with the specific sequence for which reportable knowledge was assessed. The overall number of verbalizers in the Sequence_T_ condition was significantly higher than in the Random_C_ condition, χ^2^(1, *N* = 103) = 4.17, *p* = .04. This result cannot be explained by the representational strength of the specific sequence as participants in both conditions did not practice it before the manipulation phase. The amount of training with the sequence for which verbal knowledge was assessed was exactly the same in both conditions. Thus, the type of training (random or sequenced) affected the probability of detecting the transfer sequence independently of its representation strength, possibly by causing unexpected changes in performance.

Second, a comparison of the Sequence_T_ condition with the Sequence_C_ condition seems reasonable. Based on the findings of Rünger and Frensch (2008) and the notion of unexpected events as triggers of search processes, a shift to a different sequence (i.e., Sequence_T_) could potentially lead to rates of verbalizable knowledge that are even higher than the ones obtained after continuous practice of a single sequence (i.e., Sequence_C_). However, the proportion of verbalizers in the Sequence_T_ group did not differ significantly from the overall proportion of verbalizers in the Sequence_C_ condition, χ^2^(1, *N* = 100) = 0.75, *p* = .39. It is notable though, that participants in the Sequence_T_ group acquired at least the same amount of verbalizable sequence knowledge as those in the Sequence_C_ condition despite the fact that they received less than half the amount of training with the specific sequence that was administered in the Sequence_C_ condition.

We now turn to the four conditions that crossed the factors prior sequence practice and RSI manipulation (Random_C_, Random_RSI_, Sequence_C_, Sequence_RSI_). First, the effect of the timing manipulation on the proportion of verbalizers was tested separately for the random training and sequence training conditions. While the proportion of verbalizers was numerically larger in both conditions when the RSI manipulation was present versus when it was absent, there was no significant difference; random training: χ^2^(1, *N* = 96) = 0.23, *p* = .88; sequence training: χ^2^(1, *N* = 112) = 0.36, *p* = .55. The effect of RSI remained statistically insignificant after pooling the data of the two training conditions, χ^2^(1, *N* = 208) = 0.62, *p* = .43. Therefore, we assessed the effect of training (sequence vs. random) by collapsing over the RSI conditions. Participants who practiced the systematic sequence over the whole experiment (Sequence_C_ together with Sequence_RSI_) were more likely to acquire verbalizable sequence knowledge than participants who practiced the sequence in the final 180 trials only (Random_C_ together with Random_RSI_), χ^2^(1, *N* = 208) = 3.87, *p* = .05. Thus, more sequence training led to more explicit sequence detections, but the violation of timing expectancies did not. This result supports the re-presentational strength hypothesis (but see below).

Summing up, we observed that, overall, there was more explicit sequence knowledge after sequence training as compared to random training. However, note that this effect does not have to be based on differences in sequence representation strength: When the fixed sequence was present during training, then a spontaneous search could succeed at any point in time in the experiment. During random training however, a search for task regularities could not be successful. If one assumes that at any point in time during the experiment, a search for regularities spontaneously occurred with some fixed probability (cf. Gaissmaier & Schooler, 2008), then the cumulative probability that such a search uncovered the systematic pattern by the end of the manipulation phase is higher if the regularity could be discovered in the training phase *and* in the manipulation phase (i.e., in the groups with systematic training), as opposed to the situation in which the regularity could be caught in the manipulation phase only (i.e., in the groups with random training).

### The probability of sequence detection within the manipulation phase

The results presented so far are not consistent in that neither representation strength nor unexpected events provided an unequivocal explanation. Since the last 180 trials of the experiment are critical for assessing the effects of our manipulations, we need to know the probability of detecting the systematic sequence during this final manipulation phase. Notably, in the three groups that received a regular sequence from the start, there were several verbalizers who reported in the interview that they had detected the sequence before the second half of the third block (the beginning of the manipulation phase). If we want to compare detection probabilities after a certain amount of random training and the same amount of sequence training, it is problematic to include in this analysis the verbalizers who already found the sequence during the training phase. Moreover, when considering the Sequence_T_ group, the group of verbalizers (participants who recalled the sequence of the manipulation phase) includes participants who detected the first sequence early in training and were therefore likely to search for the second sequence after transfer. It is likely that these verbalizers did not discover the second sequence due to the experimental manipulation, but because of an a priori awareness of the existence of regularities. For a fair comparison of the effects of the different training and manipulation conditions, it is necessary to focus on participants who did not develop verbalizable knowledge prior to the last 180 trials that were structured according to the same systematic sequence in all conditions.

We identified the point in time when explicit sequence knowledge occurred by adapting a method described by Haider and Rose (2007). Several results in the field of cognitive skill acquisition support the assumption that the time point in training when a sudden and unusually large decrease in RT occurs marks the point of insight into a hidden regularity that can be used to optimize task processing. Moreover, in the study by Haider and Frensch (2002), participants with an RT drop were also those who reported in the postexperimental interview that they had detected the regularity during training, whereas participants without an RT drop were not able to name the regularity. A study in which participants were interrupted and interviewed immediately after an RT drop revealed that all of these participants were able to name the regularity, independent of the number of training blocks they had completed before (Haider et al., 2005). In contrast, participants with no RT drop were not able to describe the regularity, even after the maximum amount of training.

Haider and Rose (2007) described a procedure to identify discontinuities in an RT data series that relies on median filtering (to eliminate strong oscillations) and the examination of the minimum-function of this filtered data. We applied this procedure to the RT data in the current study. For each participant, RTs were filtered with a lag-5 median filter (the first median was computed over RTs 1 to 5, the second over RTs 2 to 6, etc.), with the first four trials in each block remaining without an assigned median value. In a second step, we computed an individual minimum function of the median RTs. The value of this minimum function only changes if the present median is smaller than the last value of the minimum function. Thus, it describes the lower RT limit over the course of trials. For each participant, we defined the trial in which the minimal RT (reflected in the individual minimum function of the running RT median) fell below a predetermined level. We used 350 ms in the sequence training condition and 400 ms in the random training condition as cut-offs for the minimum function. The different cut-offs account for the between-group RT differences after random training and after sequence training (participants without explicit sequence knowledge only). Note that for participants with no verbalizable knowledge, the mean of the minimum functions in the last 60 trials of the experiment was 455.42 ms (*SD* = 61.76) after sequence training, and 480.43 (*SD* = 59.99) after random training. With the chosen RT limits we can be reasonably sure that correct responses occurring this fast after stimulus onset are extremely unlikely in the six-choice color matching task unless the upcoming response can be anticipated on the basis of explicit sequence knowledge.

Next, we analyzed how the assessment of verbalizable sequence knowledge and the subjective time point of detection corresponded with the occurrence of RT drops. The correspondence was high overall, but some exceptions occurred. Fifteen out of 20 verbalizers in the random training conditions showed an RT drop during the manipulation phase (i.e., when being exposed to the regular sequence). Five did not, probably because explicit sequence knowledge was generated near the end of the experiment and therefore did not affect task performance strongly enough to be detected in the RT analysis. Consistent with this interpretation, these participants indicated the fourth block as the block of sequence detection in the post-experimental interview. In the sequence training conditions, the experimental blocks in which the RT drops were found matched the blocks of sequence detection indicated in the interview except in the following cases: Three out of 37 verbalizers indicated Block 4 and showed no RT drop at all, or one that fell short of the 350 ms criterion. These participants were categorized as having acquired explicit sequence knowledge at the end of the experiment. One verbalizer without an RT drop indicated that he discovered the sequence in Block 2. Finally, one participant correctly reported the sequence but denied having detected the sequence during the experiment. This participant also showed no RT drop. Categorizing him as a “non-verbalizer” or as a participant who had detected the sequence in the last block did not alter the results. In the analyses below he was added to the latter category because he matched the recall criterion.

We used the RT drop to filter out those verbalizers in conditions with regular repeating sequences throughout the experiment who had detected the fixed sequence prior to the manipulation phase (12 verbalizers in the Sequence_C_ and 10 in the Sequence_RSI_ group, respectively). Filtering was also applied to the Sequence_T_ group, thereby excluding nine participants who had become aware of the first sequential regularity in the initial two and a half blocks. The analysis of the proportion of verbalizers who acquired explicit sequence knowledge during the manipulation phase of the experiment (based on a sample size corrected for the verbalizers who detected the sequence earlier) provides a direct test of the unexpected event hypothesis against the memory strength view: If a search is more likely to be triggered when there are unexpected changes in task processing, we expect a higher percentage of (new) verbalizers when the sequence changes (i.e., in the Sequence_T_ condition) as compared to the group for which it is identical with the one that was practiced before (in the Sequence_C_ condition). While the unexpected event hypothesis predicts more sequence knowledge for the Sequence_T_ as compared to the Sequence_C_ condition, the reverse prediction holds for the strength-based account. Assuming that continuous practice with a sequence strengthens the representation of this sequence until it becomes strong enough to lead to RT drops and verbalizable knowledge, one would expect that the Sequence_C_ condition yields a larger percentage of verbalizers during the manipulation phase as compared to the Sequence_T_ condition. The results, however, support the unexpected event hypothesis: The proportion of sequence detections in the last 180 trials was higher in the Sequence_T_ condition than in the Sequence_C_ condition. This difference approached significance in a one-tailed test, χ^2^(1, *N* = 80) = 2.55, *p* = .055. Thus, the proportion of sequence detections in the manipulation phase appeared to be higher, rather than lower, when participants received a *different* sequence during training than when they received the same sequence. This cannot be explained by the representational strength hypothesis without difficulties.

However, it could be argued that the comparison of the probability of sequence detection in the last 180 trials between the Sequence_C_ condition and the Sequence_T_ condition is inadequate because it involves the comparison of different time points on the learning curve for the critical sequence (late for the Sequence_C_ condition and early for the Sequence_T_ condition). We therefore also compared the number of verbalizers in the first 180 trials in the Sequence_C_ condition with the number of late verbalizers in the final 180 trials in the Sequence_T_ condition (identified by the RT-drop analysis and the interviews). We found that there were significantly fewer verbalizers in the Sequence_C_ condition than in the Sequence_T_ condition, χ^2^(1, *N* = 92) = 4.39, *p* = .04. To get a more reliable result we pooled the data of the Sequence_C_ and Sequence_RSI_ conditions that did not differ from each other in the first 180 trials and repeated the test. The difference proved to be reliable, χ^2^(1, *N* = 149) = 4.66, *p* = .03. Thus, 180 trials of training with a systematic sequence led to more participants with verbalizable knowledge if a *different* regular sequence was practiced before, even when participants who assumedly had formed explicit knowledge about this other fixed sequence were excluded from the analysis. To put it another way, comparing the two conditions that were equated for the amount of practice with the specific sequence of the manipulation phase, we found that prior exposure to a different systematic sequence facilitated the generation of explicit knowledge, which is not compatible with the representational strength hypothesis.

**Figure 2.**
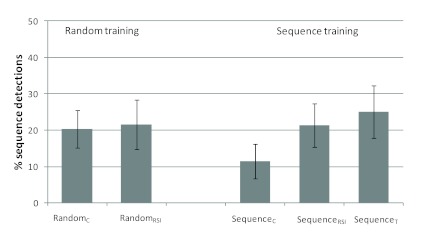
Percentage of participants who detected the sequence in the manipulation phase of the experiment (last 180 trials). Error bars represent estimated standard errors for percent values.

**Figure 3.**
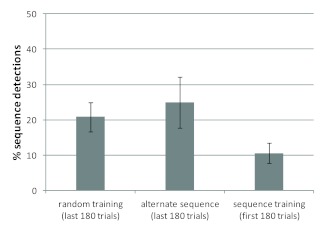
Percentage of participants who acquired their explicit knowledge within 180 trials of the first encounter with the specific sequence. Error bars represent estimated standard errors for percent values.

In light of the last finding, it seemed promising to compare the proportion of sequence detections during the first 180 trials in the sequence training conditions (pooling Sequence_C_ and Sequence_RSI_) with the proportion of sequence detections during the manipulation phase in the random training conditions (pooling Random_C_ and Random_RSI_). The comparison showed that explicit sequence knowledge was more likely to be acquired after random training than without any preceding training, that is, in the first 180 trials of the Sequence_C_ and Sequence_RSI_ groups, χ^2^(1, *N* = 209) = 4.18, *p* = .04 (see Figure 3). Sequence detection was also numerically more likely after random training than after sequence training with the same sequence (i.e., in the last 180 trials of the Random_C_ and Sequence_C_ groups), but this difference was not significant, χ^2^(1, *N* = 103) = 1.47, *p* = .23 (see Figure 2). Taken together, these results suggest that both a shift from a different systematic sequence and a shift from randomly structured trials to a target sequence seem to facilitate the acquisition of reportable sequence knowledge to some degree. This finding accords with the unexpected-event hypothesis.

Finally, we considered the influence of the timing manipulation for the data filtered for sequence detections prior to the manipulation phase. Were participants more likely to generate reportable knowledge when they experienced an unexpected speed-up in task performance? There was no effect of the RSI manipulation after random training; Random_RSI_ versus Random_C_: χ^2^(1, *N* = 96) = 0.02, *p* = .44, one-tailed (cf. Figure 2). After sequence training, there was a slight tendency towards a higher detection probability if the timing was manipulated compared to the group in which it was not manipulated; Sequence_RSI_ versus Sequence_C_: χ^2^(1, *N* = 90) = 1.74, *p* = .09 (one-tailed). Though not significant, this result might indicate that if unexpected changes in timing affect the probability of acquiring explicit know-ledge at all, then only if the representation of the specific sequence has some strength already. We return to this point in the Discussion section.

### How are free and cued verbal report related?

For exploratory purposes, the post experimental interview also contained cued recall. Mean proportion of correct triplets in the cued recall test and mean confidence ratings correlated positively (*r* = .76, *p* < .001). When 50% correct (three out of six triplets completed correctly) on the cued recall test was taken as criterion for a participant to be categorized as possessing explicit sequence knowledge, this classification correlated substantially with the verbal report classification (φ = .73, *p* <.001). While some participants were classified differently based on cued recall as compared to verbal report, an inspection of the RT data suggested that free verbal report was the better measure as it showed a closer relationship to abrupt changes in task performance (Haider & Rose, 2007). Verbalizers who did not perform well in cued recall, showed similar performance curves as verbalizers who did (i.e., RT-drop indicative of sequence detection in either case), whereas participants who achieved a high score in the cued recall test but not in free verbal report behaved more like other non-verbalizers (i.e., no RT-drop indicative of sequence detection).

### Is there implicit sequence learning in the six-choice color-matching task?

So far, we assumed that participants learned the sequential regularity of the SRT task implicitly and that a subset of participants then moved on to generate explicit sequence knowledge. In this section, we analyze participants’ RT data in order to provide evidence for implicit sequence learning. Each of the four experimental blocks was divided into two parts, resulting in eight 60-trial runs.

Only one of our experimental conditions provides a direct measure of implicit sequence knowledge in a within-subject comparison. This is the Sequence_T_ condition where we can look at the effect of a transfer sequence on performance. In the following, the Random_C_ and Sequence_C_ groups are considered first because their comparison provides some indications of implicit sequence learning, too. The data of the experimental groups with RSI deviants in the manipulation phase are not reported here because the irregularities in timing increase RT variability and obscure the already small RT-effects.

**Figure 4.**
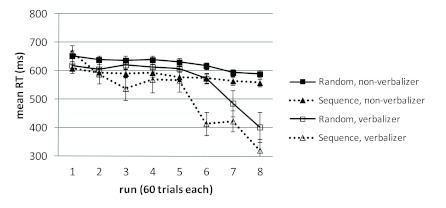
Mean reaction times (RTs) over the course of the experiment for participants of the control groups (Random_C_ and Sequence_C_), with and without explicit sequence knowledge in the postexperimental interview. Error bars represent standard errors of the mean (by group and run).

There was a more pronounced RT decrease in the Sequence_C_ group than in the Random_C_ group, indicated by a statistical interaction of Run and Training Condition, *F*(7, 791) = 3.39, *p* = .001, η^2^ .03. However, if participants categorized as verbalizers in the verbal report task were excluded from this analysis, this difference in the run effect was diminished (*F* < 1), that is, only verbalizers showed the effect, *F*(7, 189) = 4.57, *p* < .001, η^2^ .15. Thus, the larger mean improvement in the Sequence_C_ condition appears to be a result of explicit sequence knowledge affecting the performance of verbalizers. This means that the amount of implicit sequence knowledge acquired in this experiment was possibly not large enough to show up in this between-group analysis. Figure 4 shows mean RTs for verbalizers and non-verbalizers in the two training conditions.^1^

We also compared the improvement during the final 180 trials (Runs 6 to 8 in Figure 4) with the improvement during the preceding 180 trials (Runs 3 to 5 in Figure 4), separately for each training condition, in a within-subject analysis with the factors Part (final vs. preceding) and Run (1-3). Only participants without explicit know-ledge (non-verbalizers) were included in this analysis. The results suggest, at least for the Random_C_ condition, that some sequence knowledge was acquired implicitly: There was a main effect of part in the Random_C_ condition, *F*(1, 46) = 21.16, *p* < .001, η^2^ .32, and in the Sequence_C_ condition, *F*(1, 38) = 23.50, *p* < .001, η^2^ .38, as well as a main effect of run in the Random_C_ condition, *F*(2, 92) = 7.91, *p* = .001, η^2^ .15, and in the Sequence_C_ condition, *F*(2, 76) = 7.00, *p* = .002, η^2^ .16, indicating a decrease in RT over the course of training. Importantly, the was an interaction of Part and Run in the Random_C_ group, *F*(2, 92) = 3.50, *p* = .03, η^2^ .07, but not in the Sequence_C_ group, *F*(2, 76) = 1.26, *p* = .29, η^2^ .03. The interaction indicates that in the Random_C_ condition the improvement was more pronounced in the final part in which some implicit sequence knowledge could influence performance as compared to the preceding runs with randomized material. This interpretation seems plausible, given that for ordinary practice effects one would expect a decreasing rate of improvement over the course of training instead.

**Figure 5.**
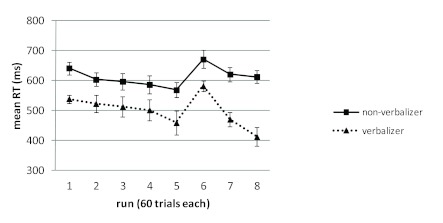
Mean reaction times (RTs) over the course of the experiment for participants with and without explicit sequence knowledge in the Sequence_T_ group (training with sequential material, transfer to an alternate sequence in Run 6). Error bars represent standard errors of the mean (by group and run).

A more effective within-subject test of implicit sequence learning is possible in the Sequence_T_ group. A within-subject ANOVA comparing RTs in the first and second half of the block in which the alternate sequence was introduced (Runs 5 and 6 in Figure 5) revealed a significant increase in RT when the repeating sequence was changed, *F*(1, 42) = 19.89, *p* < .001, η^2^ .32. This effect does not depend on explicit sequence knowledge because it was also found in participants that expressed no verbalizable sequence knowledge (interaction Transfer Effect × Verbalization: F < 1). Taken together, it is evident that implicit sequence knowledge was acquired and expressed in the six-choice color-matching task.

## Discussion

In this experiment, we studied the influence of unexpected events and implicitly acquired sequence knowledge on the likelihood that a fixed repeating sequence is (a) detected, (b) used for a pronounced improvement in task performance, and (c) verbalized in a postexperimental interview. There appears to be no simple link between the amount of practice with a specific sequence and the probability of acquiring explicit sequence knowledge. Our initial assessment of verbalizable sequence knowledge revealed that training with the systematic sequence of the manipulation phase yielded more verbalizable knowledge than training with random stimulus material, a finding that supports a strength based account of the generation of explicit sequence knowledge. However, we also observed that training with a different systematic sequence was at least as effective in producing verbalizable knowledge about the sequence of the manipulation phase as training with the same sequence.

Subsequent analyses that focused on sequence detections that occurred during the manipulation phase further weakened the strength based view. In these analyses we excluded participants who had detected a sequence prior to the manipulation phase based on a validated procedure identifying abrupt increases in performance (Haider & Rose, 2007). First, the probability of detecting the six-key FOC sequence within 30 repetitions was not higher (in fact, it was lower!) after 300 trials of training with the repeating sequence than after the same amount of training with randomized material. Second, the first 180 trials of exposure to a repeating sequence were more likely to lead to detection of the task regularity when they were presented after 300 trials of training with randomly sequenced material, than at the very beginning of the experiment. Third, detection of the repeating sequence was more likely after training with a different fixed sequence than with the same fixed sequence. Our results suggest that the generation of explicit knowledge cannot be explained exclusively on the basis of the strength of the implicitly acquired sequence representation, as it was assumed, for example, by Cleeremans (2006) and by Cleeremans and Jiménez (2002).

The impact of experimentally induced, unexpected changes in timing on the generation of verbalizable knowledge needs to be explored further to allow firm conclusions. The effect of the RSI manipulation was weak, if present at all. Furthermore, while it is plausible to interpret the effect of transfer to a different fixed sequence as mediated by participants experiencing unexpected changes in the speed of performance (cf. Rünger & Frensch, 2008), a direct proof that sequence transfer is effective via this route is still lacking.

In summary, we presented tentative evidence that inducing an unexpected RT decrease (due to a transfer from randomized to sequenced material) as well as an unexpected RT increase (due to a transfer to another sequence) resulted in a higher proportion of participants acquiring verbalizable sequence knowledge. This is in line with the prediction of the unexpected-event hypothesis that experiencing something unexpected in one’s processing of a given task calls for an explanation and thereby triggers a controlled search process within context of the task. If the task contains a regularity that participants did not notice before, the search can lead them to discover the regularity and to represent it explicitly.

One might argue that training in our experiment was quite short, and that representation strength would have had a larger effect after more sequence repetitions. That is, with more training (and a stronger sequence representation), more of the remaining nonverbalizers might have discovered the regularity. There are some details in our experimental data that speak against this possibility. The probability of sequence detection should be relatively higher in later than in earlier blocks of the experiment, simply because representation strength should, on average, be greater in later blocks. However, we found no difference between the probability of sequence detection in the first (10.6%) and in the last 180 trials (11.4%; Sequence_C_ condition). Moreover, detection probability at the end of the experiment turned out to be higher after random training and after training with another (undetected) sequence than after the same amount of training with the same (hitherto undetected) sequence. This means that detection probability was higher when the implicitly acquired representation of the target sequence must have been relatively weak. Therefore, we conclude that in the present experiment, sequence detection did not depend on the strength of the specific sequence representation. Yet, the possibility remains that representation strength affects detection probability after much more training than in our study.

In line with the unexpected-event hypothesis, the probability of detecting a sequence within 30 repetitions was higher when participants were transferred to this sequence after performing 300 trials of training with another repeating sequence (as compared to the situation/presentation/condition when the sequence did not change). This effect cannot be accounted for by representation strength of the first sequence because reportable knowledge was assessed for the unpracticed sequence of the manipulation phase. Further, in all conditions in which a repeating sequence was administered from the beginning, there were several participants who acquired explicit knowledge before the manipulation phase. This result provides a further argument against an explanation that attributes the generation of explicit sequence knowledge to representation strength alone. Participants who detected the sequence in the first or in the second experimental block were unlikely to possess a strong implicit sequence representation. It is likely that participants who detected the systematic sequence in the first blocks had an implicit sequence representation of lower strength at the time of detection compared to the representation strength in the last training block of participants who have never generated explicit sequence knowledge. All in all, the results suggest that no especially stable or strong sequence representation is required for the generation of explicit sequence knowledge. Instead, it seems crucial that a controlled search is triggered, and that the respective regularity is present in the material at that particular point in time. Potential triggers of the search need not be related to the strength of the implicit sequence representation. For instance, a feeling of unexpected fluency might be induced by (experimental) means that are unrelated to sequence learning. However, it is reasonable to assume that higher representation strength of an implicitly acquired sequence is more likely to trigger a search because it can lead to more distinct unexpected events (i.e., fast or premature but correct responses). This search can lead either to the detection of the trained sequence (if still present) or to a novel repeating sequence, if the regularity has just changed. Over the course of a sequence learning experiment (and in real life), strengthening of representations proceeds mandatorily with continued training. As participants perceive and monitor their own behavior and as implicit sequence knowledge affects task performance, representation strength should naturally be related to the effectiveness of unexpected events as triggers of a controlled search. Such an interaction would explain the way in which some participants in standard sequence learning tasks (i.e., without artificially induced unexpected events) acquire reportable sequence knowledge.

For assessing the impact of the manipulation phase on verbal sequence knowledge, we decided to exclude verbalizers that likely detected the sequence before the manipulation phase in the final 180 trials. Interestingly, all verbalizers of the Sequence_T_ group who detected the sequence present in the first 300 trials also correctly reported the second sequence they experienced in the final 180 trials. Having incidentally detected some regularity once, seems to trigger a search for new regularities, if the one first discovered does not longer apply. This transfer effect is compatible with the unexpected event hypothesis, and less so with a strength explanation (because the representation of the second sequence is weak).

Some indications of an indirect link between the strength of the implicit sequence representation and the probability of conscious detection of the sequence can also be found in our data. For example, for participants who were exposed to the timing manipulation, there was a tendency towards a higher detection probability after sequence training, but not after random training (cf. Figure 2). Apparently, training with a regular sequence made participants more sensitive to small timing deviations in their performance, possibly because these deviations violated the expectancy of smooth and speedy task performance. In line with this possibility, RTs were (numerically, not significantly) more variable in the first 60 trials of the manipulation phase than in the last 60 trials of the training phase in the Sequence_RSI_ group, which was not the case in the Sequence_C_ group. Reliable evidence for a possible interaction of this kind between implicit sequence knowledge and the effectiveness of unexpected events as triggers of search processes has been published by Haider and Frensch (2009). They showed that the insertion of computer generated (i.e., allegedly) premature correct responses increased the probability of rule detection late in training to a larger extent than during the first experimental blocks.

Over training, the development of implicit and explicit sequence knowledge might be interrelated, but we can only speculate on how a search was triggered in participants who detected the sequence very early in training. What kind of event can trigger a closer inspection of the material before much experience with the repeating response sequence has accumulated? One possible account may be found in participants’ processing style (global vs. local; cf. Navon, 1977), regulatory focus (i.e., promotion vs. prevention; Higgins, 1997), and access to higher order information. This possibility has been discussed and investigated by Förster and colleagues (e.g., Förster & Higgins, 2010; Kuschel, Förster, & Denzler, 2010). Their general idea is that when participants are approach-oriented (rather than avoidance-oriented) processing tends to become global, attention is distributed more widely, and access to higher order information (e.g., the semantic content of metaphors) is facilitated. Therefore, participants who detected the sequence early in our experiment may have processed the color SRT task more globally and for example, monitored “subjective randomness” of successive trials instead of dealing with each trial as an isolated task to be accomplished before the next trial can be undertaken. As a consequence, their expectation of randomness could have been violated early in the training phase. These ideas are in line with the unexpected-event hypothesis, but they are not directly related to accounts of the generation of verbalizable sequence knowledge that are based on implicitly acquired sequence representation strength. Individual variables such as regulatory focus (Higgins, 1997), coping style (approach vs. avoidance; cf. Carver 2006; Carver, Scheier, & Weintraub, 1989), or need for cognition (cf. Cacioppo, Petty, Feinstein, & Jarvis, 1996), as well as situational variables such as affective states (cf. Kuschel et al., 2010) are factors that should also influence the probability of sequence detection by affecting the processing style of the individual participant. While these factors are worth investigating in the future, they most likely did not influence the current results in a systematic way.

## Conclusion and Outlook

The results of the present study corroborate the notion that explicit sequence knowledge is generated if a search is triggered during task processing. We propose that the trigger for this search is an unexpected event which can, but need not, be related to the amount of preceding training or the strength of implicitly acquired sequence knowledge.

Implicit as well as explicit sequence knowledge can contribute to performance improvements in sequence learning. Usually, possessing and applying explicit knowledge about a hidden regularity speeds up performance as correct anticipations become possible. On the other hand, relying on more automatic processes (e.g., relying on implicit sequence knowledge that pre-activates responses) ensures efficient task performance (i.e., mostly fast and correct responses) without requiring substantial control resources. The decision between continuing the use of previously acquired routines and investing resources in a search for promising new regularities might be a strategic one and depend on individual preconditions, for instance, available working memory capacity. These are questions open to future research with larger samples.

## Footnotes

1 An analysis over the first five runs only (before the manipulation phase) in which the RT data of nonverbalizers of all five experimental groups receiving sequence training or randomized training were pooled revealed no Run × Training interaction, too, *F*(4, 704) = 1.10, *p* = .35.
